# Optimizing the Practice Environment for Medical Staff in the Post-pandemic Era: A Discrete Choice Experiment

**DOI:** 10.3389/fpubh.2022.911868

**Published:** 2022-07-18

**Authors:** Peilin Wu, Zhenjing Li, Wei Guo, Li Wang, Xiangxiang Chang, Yanqun Zhang, Li Wang, Lidan Wang, Qunying Liu

**Affiliations:** ^1^School of Health Services Management, Anhui Medical University, Hefei, China; ^2^Postgraduate Affairs Department of the Party Committee, Anhui Medical University, Hefei, China; ^3^Center for Health Policy Research, Anhui Medical University, Hefei, China; ^4^Medical Humanities Research Center, Anhui Medical University, Hefei, China

**Keywords:** medical staff, Chinese healthcare, job preferences, discrete choice experiment, COVID-19 pandemic

## Abstract

**Objective:**

This study aimed to elicit the stated job preferences of Chinese medical staff in the post-pandemic era and identify the relative importance of different factors in the practice environment.

**Methods:**

We used an online discrete choice experiment (DCE) survey instrument to elicit the job preferences of medical staff (doctors and nurses) in tertiary hospitals in Anhui, China. Attributes and levels were generated using qualitative methods, and four attributes were considered: career development, workload, respect from society, and monthly income. A set of profiles was created using a D-efficient design. The data were analyzed considering potential preference heterogeneity, using the conditional logit model and the latent class logit (LCL) model.

**Results:**

A total of 789 valid questionnaires were included in the analysis, with an effective response rate of 73.33%. Career development, workload, respect from society, and monthly income were significant factors that influenced job preferences. Three classes were identified based on the LCL model, and preference heterogeneity among different medical staff was demonstrated. Class 1 (16.17%) and Class 2 (43.51%) valued respect from society most, whereas Class 3 (40.32%) prioritized monthly income. We found that when respect from society was raised to a satisfactory level (50–75% positive reviews), the probability of medical staff choosing a certain job increased by 69.9%.

**Conclusion:**

Respect from society was the most preferred attribute, while workload, monthly income, and career development were all key factors in the medical staff's job choices. The heterogeneity of the medical professionals' preferences shows that effective policy interventions should be customized to accommodate these drive preferences.

## Introduction

The COVID-19 pandemic constitutes an international public health emergency (1). Frontline medical staff continue to play a critical role in effectively controlling its spread in China. Medical staff are occupied with the treatment of patients, epidemiological investigation, isolation, and observation of close contacts (2). In the early stages of the pandemic, the shortage of personal protective equipment (PPE) necessitated that medical staff work continuously for at least 6 h while wearing protective clothing (3). Multiple studies of the COVID-19 pandemic have shown that medical staff are more prone to mental health issues, such as anxiety and depression, due to the continuous impact of the pandemic, occupational exposure, heavy workload, insufficient PPE, and doctor-patient conflicts (4–6). Such negative states could affect the quality of their work, reduce job satisfaction, and even lead to job burnout (7–9).

Currently, research on job satisfaction of medical staff in China can be divided into two categories: research on influential factors and status investigation. Research on influential factors has suggested that factors affecting job satisfaction among medical staff are mainly due to their poor practice environment, such as low income, high work pressure, workplace violence, unfair promotions, low social status, etc. (10–14). However, the analyses of these influencing factors do not reveal information on the trade-offs of these practice environment factors by medical staff. Meanwhile, the results from status investigation found that medical staff in tertiary hospitals have lower job satisfaction compared to those working in primary hospitals and are more likely to experience burnout (15). Medical staff in tertiary hospitals, in both numbers and service capabilities, constitute the primary resources of China's medical and health systems (16). Therefore, improving work conditions and incentives for medical staff in tertiary public hospitals requires immediate attention.

To improve job satisfaction among medical staff, the Chinese government has repeatedly emphasized the creation of a sound atmosphere of “*Zun Yi Zhong Wei*” in society in terms of salary, career development, social status, and so forth (17). This implies that the Chinese government is trying to improve the practice environment of medical staff, to increase respect for their professional and social status. But as mentioned above, the existing research results have not been able to provide more reference information for government departments, such as how do medical staff make trade-offs among these practice environment factors?

The discrete choice experiment (DCE), an econometric method based on random utility theory and formulated from the selected model, has found wide use in the field of human resources for health (HRH) research in recent years (18–20). However, previous studies using DCEs have primarily focused on solving the problem of human resource shortages for health in low- and middle-income countries and remote areas (21, 22). In the field of HRH research in China, the application of DCE is mainly focused on recruitment and retention of medical staff in rural areas (23), while the incentive preferences of medical staff in tertiary hospitals is rarely considered (24). In addition, no such DCE studies have been conducted since the pandemic outbreak.

Therefore, the present study conducted a DCE in tertiary hospitals. We aimed to answer the following questions in the context of developing a good social atmosphere based on “*Zun Yi Zhong Wei*” and emergence of the COVID-19 pandemic: (a) What factors are considered by medical staff in tertiary hospitals when balancing different job options? (b) What is the relative importance (RI) of these factors? (c) What are the different preferences of different subgroups of medical staff? The answers to these questions will be of great significance to the Chinese government in optimizing the practice environment for medical staff in the post-pandemic era and in providing incentives to enhance their enthusiasm for work.

## Methods

### Discrete Choice Experiments Design

A series of qualitative studies were conducted to determine job attributes and their corresponding levels (25, 26). First, we systematically reviewed national and international journal articles on the application of DCEs in HRH research (27–30). Second, to select the most important attributes to assess practice environment and levels to measure these attributes in our study and make its design more accurate, five policymakers and ten medical staff (five doctors and five nurses) were invited to conduct a semi-structured interview. On the basis of the literature review and interview results, we categorized training and development opportunities under “career development attributes,” and categorized working hours and work intensity under “workload attributes.” Finally, four attributes were included in this study. Table 1 shows a detailed explanation of the attributes and their corresponding levels (31, 32).

**Table 1:** Detailed explanations of attributes and levels.

**Attributes**	**Explanations**	**Level**
Career development	Career development includes opportunities for professional training, networking, and promotion.	➢ Insufficient ➢ Sufficient
Workload	Workload refers to the number of hours worked in a week by medical staff	➢Light (40 h/week) ➢ Medium (40–48 h/week) ➢ Heavy (48–56 h/week) ➢ Maximum (More than 56 h/week)
Respect from society	Respect from society refers to the evaluation, recognition, and respect given to medical staff by the public or in public opinion.	➢ Poor (25% or less of the public give positive evaluations) ➢ Normal (25–50% of the public give positive reviews) ➢ Good (50–75% of the public give positive reviews) ➢ Excellent (More than 75% of the public give positive reviews)
Monthly income^a^	Monthly income includes salary, bonus, and welfare benefits (pension scheme, basic medical insurance, unemployment insurance, employment injury insurance, maternity insurance, and housing accumulation funds).	➢ 1*Average national salary (1,091 USD) ➢ 2*Average national salary (2,183 USD) ➢ 3*Average national salary (3,274 USD) ➢ 4*Average national salary (4,366 USD)

^a^*In 2019, the average annual salary of employees in urban non-private units in China was 90,501 CNY (31); the average annual exchange rate between USD and CNY in 2019 was USD 1 = CNY 6.908 (32) (Organization for Economic Co-operation and Development data. Available online: (accessed March 21, 2020)*.

Given the attributes and levels selected, 128 (4^3^ × 2^1^) pairs of job choice sets were possible. Considering the cognitive burden of the respondents and the time costs (33), a fractional factorial design was used to select a fraction of the total job choice sets to be presented to participants. Moreover, the D-efficiency was optimized based on the information obtained from the pilot study to maximize level balance and orthogonality, and minimize overlap between attribute levels.

In this study, we designed the choice set using Ngene software (version 1.1.2, Choice Metrics) and determined eight pairs of choice sets, each of which included two job options and an “opt-out” option. In each choice set, respondents had a choice between Job A and B; they also had the choice of opting out when they felt that both Job A and B could not meet their expectations. In the opt-out option, the attribute level was set to zero.

The first choice set answered by respondents was an additional choice set that was used as the rational test choice set, in which the monthly income of Job B is much higher than that of Job A while the other three attribute levels remained the same. For utility maximization, the respondent as a rational economic individual would choose Job B. Otherwise, they were identified as lacking ability to understand the DCE questionnaire, and their data would not be included in the final statistical analysis.

Prior to the formal survey, 20 medical staff members were invited to the pilot study. We made minor wording-related adjustments based on their feedback, to improved and finalize the questionnaire. Due to the impact of COVID-2019, we have chosen an online survey instead of face-to-face survey. Supplementary Figure 1 shows an example of a DCE choice set.

### Data Collection

This study was conducted in Hefei, Anhui Province, a city located in the middle of the Yangtze River Delta region, and the results of the study have certain implications for the region and its surrounding cities. We selected sample hospitals among all tertiary hospital in Hefei and considered the following inclusion criteria: it must be a comprehensive tertiary hospital; the number of hospital beds is at the top of the same level of hospitals; it mainly treats patients from the province and surrounding areas. Three hospitals were finally included. The regular staff of each clinical departments of the three sample hospitals who voluntarily participated in this study were regarded as our sample.

To ensure the quality of the questionnaire, we first took the assistance of the hospital's administrative department to establish a WeChat group with medical staff willing to participate in the survey. The research team members shared the prepared questionnaire user manual in the WeChat group, with the key points of completing the questionnaire, marked in the manual. Simultaneously, we introduced our research background, choice sets, and selection methods in the WeChat group and answered their queries. We administered the questionnaire through the Wen Juan Xing platform, and medical staff could voluntarily end its completion. Except for the DCE data, we also collected the demographic characteristics, such as gender, age, and marital status of the respondents.

Data were collected from July to August 2020. Finally, 1,076 respondents completed the DCE questionnaire. Due to differences between online and face-to-face surveys, we could not assess respondents' comprehension of the questionnaire in real time. For those who did not understand how to complete the DCE questionnaire, we could not end the survey on time. Therefore, after data collection was completed, we set strict quality control standards. A piece of data was excluded if it met one of the following criteria: (1) Choosing a single option from beginning to end (85 items deleted), (2) the time to complete the questionnaire was <180 s (64 items deleted), and (3) failing to pass the rationality test (138 items deleted). Based on these criteria, 789 valid questionnaires were retained, and the effective response rate was 73.33%, meeting the requirements for DCE data analysis (34). The study was approved by the Ethics Committee of Anhui Medical University (No: 2020H011).

### Statistical Analysis

Analysis of the DCE data was based on the random utility theory framework, where the utility that respondent *i* derives from choosing alternative *j*, in choice set *t*, is given by


(1) $$\begin{array}{r} U_{ijt}=X_{ijt}\beta +\varepsilon_{ijt};i\;=1,\ldots ,789;j\;=1,2,3;t\;=1,\ldots ,8; \end{array}$$

where β is a vector of coefficients, and *X*_*ijt*_ is a vector of variables representing the attributes of alternative *j*. For the unforced choice model, *j* =1, 2, 3. When the random term ε_*ijt*_ is assumed to be independently and identically distributed according to the Type I extreme value distribution, the model becomes the conditional logit (CLOGIT).

Based on the results of the CLOGIT model, we further explored the Mixed Logit model (Supplementary Table 1) and the Latent Class Logit (LCL) model for data analysis. Based on the goodness of fit of the model, we finally chose the LCL model (35), which assumes that there are Z distinct classes of preference parameters, β = (β_1_, …, β_z_). If respondent *i* is in class β, the utility *U*_*ij*_ of person *i* that is associated with choosing alternative *j* is


(2) $$\begin{array}{r} U_{ij|c}=\beta c,X_{ij}+\varepsilon_{ij} \end{array}$$

The LCL models in the study were estimated using “lclogit2” in Stata (version 16, Stata Corp) (36, 37). The number of classes was determined based on the Bayesian Information Criterion (BIC) (38). We first estimated preference coefficients for each class, and then produced their weighted averages over classes as the overall population means. A positive regression coefficient suggested that respondents preferred an increased value for an attribute, whereas a negative coefficient suggested that respondents preferred a decreased value for an attribute. Statistical significance was set at α = 0.05 following a two-tailed test. The attribute “monthly income” was analyzed as a continuous variable, other attributes were included as dummy variables, and we derived willingness to pay (WTP) as the negative ratio of the coefficients from other attributes to the coefficient of the “monthly income” attribute (39). The RI of attributes is calculated by dividing the difference in utility between the highest and lowest levels of a single attribute by the sum of the differences for all attributes (40).

Based on the conditional logit model, the proportion of medical staff who would choose job *i* over all other jobs available to them was calculated by


(3) $$\begin{array}{r} P_{i}=\frac{e^{\alpha_{1}+\beta_{1}x_{1i}+\beta_{2}x_{2i}+\ldots +\beta_{m}x_{mi}}}{\underset{J}{\sum }e^{\alpha_{1}+\beta_{1}x_{1j}+\beta_{2}x_{2j}+\ldots +\beta_{m}x_{mj}}}\;i,j\in J \end{array}$$

Equation (3) can then be used for a scenario analysis to explore changes in the probability of medical staff choosing a certain job under different practice environments, and to provide a more refined reference for decisions taken by government departments.

## Results

### Demographic Information

The percentage of women among the total number of participants was 85.04%. Of the participants, 91.25% were younger than 40 years old and 66.29% were nurses. The ratio of doctors to nurses was ~1:2. Further, 76.3% had a bachelor's degree, and 45.88% of them had worked for ≤ 5 years. Supplementary Table 2 shows the other included characteristics of the participants.

### Mean Preference

CLOGIT analysis results (Supplementary Table 3) were consistent with our expectations: medical staff preferred better career development, more respect from society, and higher monthly income over heavy workloads. All attribute-level preference regression coefficients were statistically significant and had a linear relationship with their changes. On the basis of this result, we selected the LCL model to further elicit the preferences of different subgroups. According to the BIC, the three-class LCL model was determined, in which Class 1 accounts for 16.17%, Class 2 accounts for 43.51%, and Class 3 accounts for 40.32%.

Overall, the LCL results (Table 2) indicated that medical staff preferred better respect from society and higher monthly income over heavy workloads. For specific attributes, medical staff mostly preferred the “good” level (50-75% of the public give positive reviews) for the respect from society, and only “workload” did not show a preference for the maximum level (more than 56 h/week). The analysis of WTP (Table 3) reveals that medical staff were willing to sacrifice three times the average national salary to obtain sufficient career development. When the workload increased from light (40 h/week) to maximum (more than 56 h/week), they expect to be paid an additional three times the average national salary based on their existing level. In terms of poor respect from society (25% or less of the public give positive evaluations), medical staff were willing to sacrifice 4–5 times the average national salary to obtain normal (25–50% of the public give positive reviews), good (50–75% of the public give positive reviews), and excellent (more than 75% of the public give positive reviews) respect from society, respectively. The results of RI (Table 2) showed that medical staff valued respect from society the most, followed by workload, monthly income, and career development prospects.

**Table 2:** The three-class latent-class-logit model estimates.

		**Class 1: prospect minded**			**Class 2: workload unconcerned**			**Class 3: income dependent**			**Mean** ^**b**^		
		**Est**	**SE**	**RI (rank)**	**Est**	**SE**	**RI (rank)**	**Est**	**SE**	**RI (rank)**	**Est**	**SE**	**RI (rank)**
**Share**		0.162***	0.015		0.435***	0.036		0.403***	0.036				
**ASC 1**		−3.975***	0.382		1.852***	0.451		−4.750*	1.844		−1.752*	0.737	
**ASC 2**		−4.162***	0.374		1.911***	0.456		−6.740*	2.627		−2.559*	1.05	
**Career development**	**Insufficient** ^**a**^			0.25 (2)			0.24 (2)			0.03 (4)			0.16 (4)
	**Sufficient**	1.947***	0.173		0.864***	0.053		−0.740	0.996		0.392	0.408	
**Workload**	**Light** ^**a**^			0.25 (3)			0.17 (3)			0.34 (2)			0.25 (2)
	**Medium**	−0.230	0.200		−0.008	0.074		0.104	0.562		0.001	0.218	
	**Heavy**	−0.514**	0.198		0.135	0.498		2.296	1.662		0.901	0.686	
	**Maximum**	−1.940***	0.250		−0.452	0.495		−5.296**	1.829		−2.645***	0.767	
**Respect from society**	**Poor** ^**a**^			0.35 (1)			0.43 (1)			0.27 (3)			0.36 (1)
	**Normal**	2.304***	0.248		0.938***	0.088		3.363***	0.860		2.136***	0.344	
	**Good**	2.515***	0.300		1.307*	0.513		6.140***	1.859		3.451***	0.781	
	**Excellent**	2.754***	0.301		1.519**	0.513		2.268*	1.092		2.020***	0.488	
**Monthly income**		0.377***	0.967	0.15 (4)	0.186***	0.030	0.16 (4)	2.642**	0.846	0.35 (1)	1.207***	0.338	0.24 (3)
**Number of participants**								789					
**Number of observations**								18,936					
**Log likelihood**								−4,073.69					
**Akaike's Information Criterion (AIC)**								8,211.38					
**Bayesian Information Criterion (BIC)**								8,462.54					

^a^ reference; ^b^ weighted average of coefficients over three classes. ***p < 0.005; **p < 0.01; *p < 0.05.

*ASC, alternative specific constant; Est, Estimate; SE, standard error*.

**Table 3:** Class specific marginal willingness-to-pay estimates.

		**Class 1: prospect minded**		**Class 2: workload unconcerned**		**Class 3: income dependent**		**Mean** ^**a**^	
		**Est**	**95% CI**	**Est**	**95% CI**	**Est**	**95% CI**	**Est**	**95% CI**
**Career development**	**Insufficient** ** → Sufficient**	−5.157***	(3.195, 7.119)	−4.640***	(3.101, 6.179)	0.280	(−0.882, 0.322)	−2.740***	(−3.383, −2.097)
**Workload**	**Light** ** → Medium**	0.609	(−7.286, −2.995)	0.043	(−7.699, 2.846)	−0.039	(2.490, −1.519)	0.101	(0.408, 4.982)
	**Light** ** → Heavy**	1.362*	(−2.452, −0.273)	−0.727	(−4.504, 5.958)	−0.869**	(0.137, 1.601)	−0.446	(−2.687, 1.794)
	**Light** ** → Maximum**	5.140***	(−1.670, 0.452)	2.426	(−0.822, 0.737)	2.004***	(−0.377, 0.456)	2.695*	(−0.261, 0.463)
**Respect from society**	**Poor** ** → Normal**	−6.103***	(3.984, 8.223)	−5.038***	(3.472, 6.604)	−1.273***	(1.069, 1.477)	−3.692***	(−4.386, −2.998)
	**Poor** ** → Good**	−6.663***	(4.074, 9.251)	−7.022*	(1.587, 12.457)	−2.324***	(1.690, 2.957)	−5.070***	(−4.386, −2.998)
	**Poor** ** → Excellent**	−7.296***	(4.609, 9.983)	−8.159**	(2.669, 13.649)	−0.858	(−0.392, 2.108)	−5.076***	(−7.421, −2.718)

^a^* weighted average of coefficients over three classes*.

*Est, Estimate; CI, confidence interval*.

### Preference in Each Class

The preferences for respect from society in different subgroups were found to be consistent and the subgroups were named based on their inter-group preference heterogeneity. Class 1 was named prospect minded class, Class 2 was named workload unconcerned class, and Class 3 was named income dependent class. The WTP for the attribute level varied greatly among subgroups. In terms of RI, for the prospect minded class, respect from society is the most important, followed by career development. For the workload unconcerned class, respect from society is the most important, and monthly income is the least important. For the income dependent class, monthly income is the most important, followed by respect from society.

### Membership Analysis

The LCL model reveals the heterogeneity of preferences among the three classes. In this section, we performed a posteriori analysis and presented a profile of medical staff in each class with a mean marginal effect (Supplementary Tables 4, 5). The results showed that gender, position, age, marital status, educational background, professional title, and working years significantly predicted class membership.

**Figure 1:**
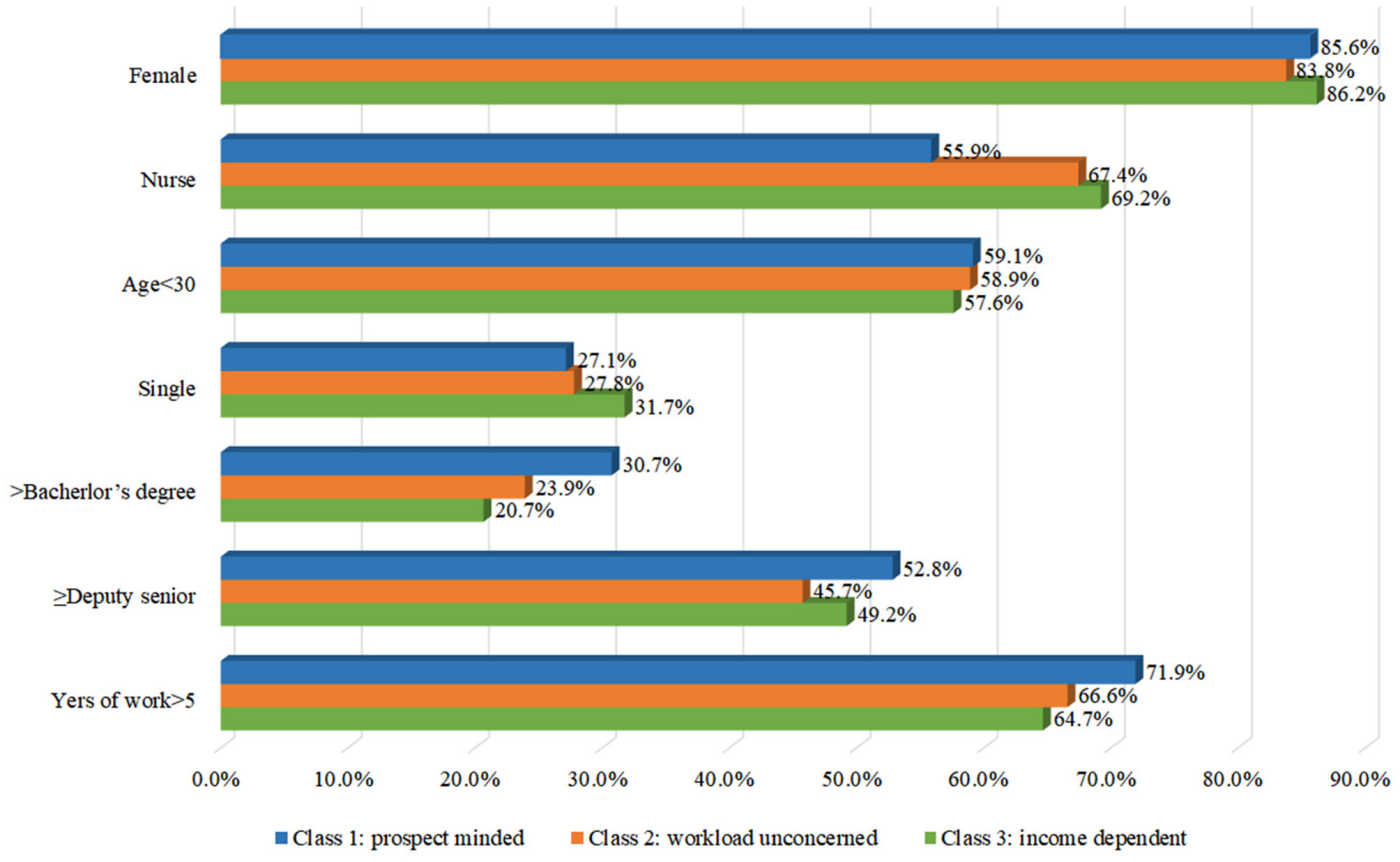
The profile of membership.

We estimated the expected values of the significant predictors, as shown in Figure 1. The prospect minded class included more doctors, and staff with higher levels of education, deputy senior titles or above and longer working years, compared with other classes. The income dependent class had the highest number of nurses, but also included more member with lower levels of education and fewer working years. For the workload unconcerned class, the proportion of medical staff with deputy senior titles or above was the smallest.

### Scenario Analysis

In the scenario analysis, we simulated changes in the job choices of medical staff under different practice environments. The results are shown in Table 4. The baseline was set as: (1) the monthly income is 2 × average national salary; (2) heavy workload (including maximum level, that is, more than 48 h/week); (3) insufficient career development; and (4) normal respect from society (including poor level, that is, <50% of the public give positive reviews).

**Table 4:** Job choice scenario analysis.

	**Career development**	**Monthly income**	**Workload ^**a**^**	**Respect from the society ^**b**^**	**Changes in the probability of selection**
Baseline	Insufficient	2*Average national salary	Heavy	Normal	–
Scenario-1	Sufficient	2*Average national salary	Heavy	Normal	+41.50%
Scenario-2	Insufficient	3*Average national salary	Heavy	Normal	+21.80%
Scenario-3	Insufficient	4*Average national salary	Heavy	Normal	+41.70%
Scenario-4	Insufficient	2*Average national salary	Medium	Normal	+47.20%
Scenario-5	Insufficient	2*Average national salary	Light	Normal	+53.20%
Scenario-6	Insufficient	2*Average national salary	Heavy	Good	+60.00%
Scenario-7	Insufficient	2*Average national salary	Heavy	Excellent	+69.90%

^a^* workload redefined into three levels: light (40 h/week), medium (40–48 h/week), and heavy (more than 48 h/week); ^b^ respect from the society redefined into three levels: normal (<50% of the public give positive reviews), good (50–75% of the public give positive reviews), and excellent (more than 75% of the public give positive reviews)*.

Compared with the baseline, when the career development changed from insufficient to sufficient, the probability of choosing a certain job increased by 41.5%; when the monthly income increased to four times the average national salary, the probability of choosing a certain job increased by 41.7%; when the workload was reduced to medium or light, it the values increased by 47.2 and 53.2%, respectively; and finally, when respect from society improved to good and excellent, the values increased by 60.0 and 69.9%, respectively.

## Discussion

In this study, we used the DCE method to elicit the job preferences of medical staff in tertiary hospitals in the post-pandemic era. We found that medical staff prefer better respect from society, higher incomes, sufficient career development, and lower workloads, which is consistent with the findings of the only two DCE studies conducted at tertiary hospitals in China (24, 41). However, our study is different in that it includes both respect from society and workload, which are highly regarded by medical staff during the interview process, and we found that medical staff appeared to be more tolerant of higher workloads after the pandemic outbreak. These research findings may help policymakers design correct policies, while optimizing the practice environment and job satisfaction of medical staff, to create a good atmosphere of “*Zun Yi Zhong Wei*.”

We found that respect from society is the attribute most valued by medical staff, which is similar to the result of the previous DCE survey conducted in Xinjiang. However, the sample of the previous study was only from senior professionals (including doctors, nurses, medical technicians and administrative staff) at a tertiary hospital (41). Moreover, we found that optimizing respect from society, which can increase to more than 60%, determines significant increases in the probability of medical staff job choices than other factors. This may be related to the fact that over 60% of medical staff have experienced doctor-patient conflict and incidents of violent injuries in China (42, 43). There is consensus among medical staff that their social status has lowered and they are not given the respect they deserve (44, 45). This has a specific relationship with the imperfect legal protection available in the Chinese health sector (46). Therefore, to improve respect from society for medical staff, it is necessary to strengthen the legislative protection of their rights and interests, as well as supervision and management through the process of law enforcement. Second, guidance and supervision of the media should be strengthened to ensure objective and fair reportage of medical matters and improve doctor-patient relationships. In addition, it is necessary to strengthen health education through courses, public lectures, and media publicity to improve the health literacy of the general public, to help them have more reasonable expectations from medical service technology and effects.

Additionally, our results showed that medical staff preferred a higher monthly income. However, in general, monthly income was not the top priority, ranking third in RI. Male doctors in China earned 78,703 RMB, and females 73,294 RMB in 2017, which were 1.28 and 1.19 times the average national salary that year, respectively (43). The average income of medical staff in the United States in 2015 was 5.9 times the average national salary, that in the United Kingdom was 2.4 times, and that in Japan was 2.8 times (47). The low pay of Chinese medical staff also contributes to the general dissatisfaction with their income (48). From one perspective, in Chinese culture, a lower income level corresponds to a lower social status (43). From another perspective, employees may perceive that a lower income level does not match their actual performance (49). In China, the income of medical staff is composed of fixed and non-fixed parts. In 2019, the fixed income of medical staff in tertiary hospitals accounted for 35.9% of the total income, whereas the non-fixed part accounted for 64.1% (50). The excessive proportion of non-fixed income leads to undesirable outcomes, such as induced demand and excessive medical care (51). Therefore, we recommend that the proportion of fixed income be increased to improve the overall income level. In addition, the existing performance-based income system needs improvement, and the value of services from medical staff should be better reflected in the non-fixed income assessment. Moreover, the effect of increasing monthly income on the probability of job selection was also at the bottom: only 21.8 and 41.7% when the monthly income increased from 2 times the average national salary to 3 and 4 times, respectively. This suggests that economic and non-economic incentives be combined when policy incentives are adopted in the future.

Regarding workload, the results revealed that most of the participants did not prefer the maximum level (more than 56 h/week) of workload. However, they were willing to accept a certain intensity of workload compared with the maximum workload. Moreover, we find that workload has no effect on the job preference of the workload unconcerned class. This finding is slightly different from the previous study, which showed that medical staff disliked workloads over 50 h per week (24). It may be that the continuous high-intensity workload during the pandemic has increased the tolerance of medical stuff to higher workload. In 2017, the average working time for medical staff in China's tertiary hospitals was ~51 h/week (43), which is why some of them believed that their income did not match their efforts. Moreover, the previous study has shown that they are least satisfied with frequently working overtime at night (10). This suggests that the hospital management department should optimize the human resource allocation of each department and improve the efficiency of personnel rotation. At the same time, qualified hospitals must consider establishing gyms and psychological consultation rooms to help medical staff manage their stress.

Further, except for the income dependent class, the other two groups showed a preference for sufficient career development. The results of the membership analysis revealed that higher educated medical staff and doctors with senior professional titles dominated the prospect minded class. They had a higher demand for respect and self-actualization, that is, better respect from society and sufficient career development. The characteristics of the workload unconcerned class are not easily evident, but it contained the lowest proportion of medical staff with deputy senior titles or above, that is, this group of medical staff is in the rising period of their careers, and promotion is their biggest appeal. However, nurses and less educated and unexperienced staff dominated the income dependent class. This may be because they had just started the job and valued monthly income more than other aspects (52). Therefore, policymakers must fully consider the preference heterogeneity of classes while designing policies.

Finally, the alternative specific constants (ASC) are close, that is, respondents' choices between Job A and B are balanced. However, they are negative, indicating that in the context of this study, respondents were more inclined to not engage in jobs, especially in the income dependent class. Further analysis found that there were two choice sets with poor respect from society or maximum workload, and respondents opted out of these two choice sets far more than the other choice sets. This further demonstrates the importance that medical staff place on respect from society and workload.

## Limitations

There are three limitations in our study. Firstly, we could not include certain attributes and levels in this study because of the degree of understanding of respondents and the method of completing the online questionnaire. Secondly, due to the impact of the COVID-19 pandemic, we adopted an online survey, which is a challenge to data quality. Although we set strict quality control criteria, it can be seen that the ASC is negative, that is, the final included sample respondent is more likely to choose opt-out. However, in the sensitivity analysis, we analyzed the choice data of all respondents (1,076) based on the conditional logit model, and the result (Supplementary Table 6) showed that the preferences eventually tended to be consistent, which means that the results reported in this study are stable and reliable. Thirdly, since the DCE is a hypothetical exercise, medical staff's actual behavior may differ depending on the context. Moreover, the monthly income attribute included in this study has a wide range of levels, which has certain limitations on the generalizability of the results.

## Conclusion

Overall, respect from society was the preferred attribute, but workload, monthly income, and career development were the key factors in the different medical staff's job choices. Policymakers should pay more attention to non-material incentives, rather than to material incentives. On the basis of the heterogeneity of medical staff's preferences for work, decision-makers should adopt different strategies for medical staff with different education levels, professional titles, and working years.

## Data Availability Statement

The original contributions presented in the study are included in the article/Supplementary Material, further inquiries can be directed to the corresponding author/s.

## Ethics Statement

The studies involving human participants were reviewed and approved by the Ethical Committee of Anhui Medical University. Written informed consent for participation was not required for this study in accordance with the national legislation and the institutional requirements.

## Author Contributions

LidW designed the study jointly with ZL. WG, LW (1st author), and XC conducted the survey supported by ZL and LW (2nd author). PW analyzed the data with support from YZ. QL was responsible for project administration. PW and LidW wrote the manuscript. LZ helped to revise the paper. All authors contributed to the article and approved the submitted version.

## Funding

This work has been funded by NSSFC (The National Social Science Fund of China) Project of Marxism and Scientific Socialism (Grant No. 19BKS169).

## Conflict of Interest

The authors declare that the research was conducted in the absence of any commercial or financial relationships that could be construed as a potential conflict of interest.

## Publisher's Note

All claims expressed in this article are solely those of the authors and do not necessarily represent those of their affiliated organizations, or those of the publisher, the editors and the reviewers. Any product that may be evaluated in this article, or claim that may be made by its manufacturer, is not guaranteed or endorsed by the publisher.
