# Supplementary material for: Optimizing the Practice Environment for Medical Staff in the Post-pandemic Era: A Discrete Choice Experiment
Source: Front Public Health. 2022 Jul 18;10:911868. doi: 10.3389/fpubh.2022.911868 (PMC9340264; doi:10.3389/fpubh.2022.911868)
Supplement: Supplementary file 1 [file Data_Sheet_1.pdf]

Supplementary Material

1 Supplementary Tables

Supplementary Table 1. Preferences of health workers based on mixed logit model

			Est	SE
ASC1			-1.643***	0.171
ASC2			-1.702***	0.174
Career development	Insufficient [a]			
	Sufficient		1.522***	0.092
Workload	Light [a]			
	Medium		-0.293***	0.075
	Heavy		-0.129	0.128
	Maximum		-1.846***	0.156
Respect from society	Poor [a]			
	Normal		2.334***	0.131
	Good		2.016***	0.147
	Excellent		2.495***	0.155
Monthly income			-1.114***	0.087
Number of participants		789		
Number of observations		18936		
Log likelihood		-4576.945		
AIC		8489.679		
BIC		9646.655		

Note: [a] reference.

ASC: Alternative specific constant; Est: Estimate; SE: Standard error; AIC: Akaike's Information Criterion; BIC: Bayesian Information Criterion

*** $p<0.01$; ** $p<0.05$; * $p<0.1$

Supplementary Table 2. Demographic characteristics of the respondents

		**N**	**%**
Sex	Male	118	14.96
	Female	671	85.04
Age (years)	≤30	327	41.44
	31-40	393	49.81
	≥41	69	8.75
Occupation	Doctor	266	33.71
	Nurse	523	66.29
Marital status	Married	558	70.72
	Other [a]	231	29.28
Education	≤ Bachelor’s degree	602	76.30
	≥ Graduate degree	187	23.70
Professional title	Primary	408	51.71
	≥ Deputy senior	381	48.29
Years of work	≤5	362	45.88
	6-10	236	29.91
	≥11	290	36.76

Note: [a] Other marital status includes unmarried, deceased spouse, divorced, and others.

Supplementary Table 3. Preferences of health workers based on conditional logit model

			Est	SE
ASC1			-0.637***	0.145
ASC2			-0.703***	0.147
Career development	Insufficient [a]			
	Sufficient		0.887***	0.045
Workload	Light [a]			
	Medium		-0.158***	0.044
	Heavy		-0.364***	0.092
	Maximum		-1.219***	0.101
Respect from society	Poor [a]			
	Normal		1.312***	0.067
	Good		1.421***	0.102
	Excellent		1.767***	0.106
Monthly income			0.445***	0.019
Number of participants		789		
Number of observations		18864		
Log likelihood		-4513.7463		

Note: [a] reference.

ASC: Alternative specific constant; Est: Estimate; SE: Standard error.

*** p<0.01; ** p<0.05; * p<0.1

Supplementary Table 4. Variables of class characterizations

**Variables**	**Variable assignment**
Gender	0 = Male [a]
	1 = Female
Occupation	0 = Doctor [a]
	1 = Nurse
Age	0 = 30 years old and above [a]
	1 = under 30 years old
Marital status	0 = Married [a]
	1 = Single
Educational background	0 = Bachelor’s degree and below [a]
	1 = More than Bachelor’s degree
Professional title	0 = Primary [a]
	1 = Deputy senior and above
Years of work	0 = 4 years and below [a]
	1= More than 5 years

Note: [a] reference.

Supplementary Table 5. Class membership: average marginal effects

	Class1: prospect minded		Class2: workload unconcerned		Class3: income dependent	
	Est	Std	Est	Std	Est	Std
Female	0.077***	0.006	0.000	0.012	-0.077***	0.012
Nurse	-0.107***	0.008	0.002	0.012	0.105***	0.011
Age<30	-0.125***	0.011	0.014	0.013	0.111***	0.012
Single	0.016**	0.008	0.042***	0.011	-0.059***	0.01
>Bachelor's degree	0.018**	0.008	-0.103***	0.012	0.085***	0.013
≥Deputy senior	0.060***	0.008	0.097***	0.011	-0.157***	0.01
Years of work>5	0.102***	0.008	-0.092***	0.012	-0.011	0.011

Note: Reference: Gender = male; Occupation = doctor; Age ≥ 30; Marital status = married; Educational background ≤ bachelor's degree; Professional title = primary; Years of work ≤ 5. Est: Estimate; SE: Standard error.

*** p<0.01;** p<0.05;* p<0.1

Supplementary Table 6. Sensitivity analysis based on conditional logit model

			**Est**	**SE**
ASC1			-0.014*	0.114
ASC2			-0.200*	0.113
Career development	Insufficient [a]			
	Sufficient		0.671***	0.032
Workload	Light [a]			
	Medium		-0.119***	0.030
	Heavy		-0.193***	0.063
	Maximum		-0.757***	0.071
Respect from society	Poor [a]			
	Normal		0.919***	0.043
	Good		0.895***	0.070
	Excellent		1.146***	0.073
Monthly income			0.445***	0.014
Number of participants		1076		
Number of observations		25824		
Log likelihood		-6952.901		

Note: [a] reference.

ASC: Alternative specific constant; Est: Estimate; SE: Standard error.

*** $p<0.01$; ** $p<0.05$; * $p<0.1$

2 Supplementary Figures

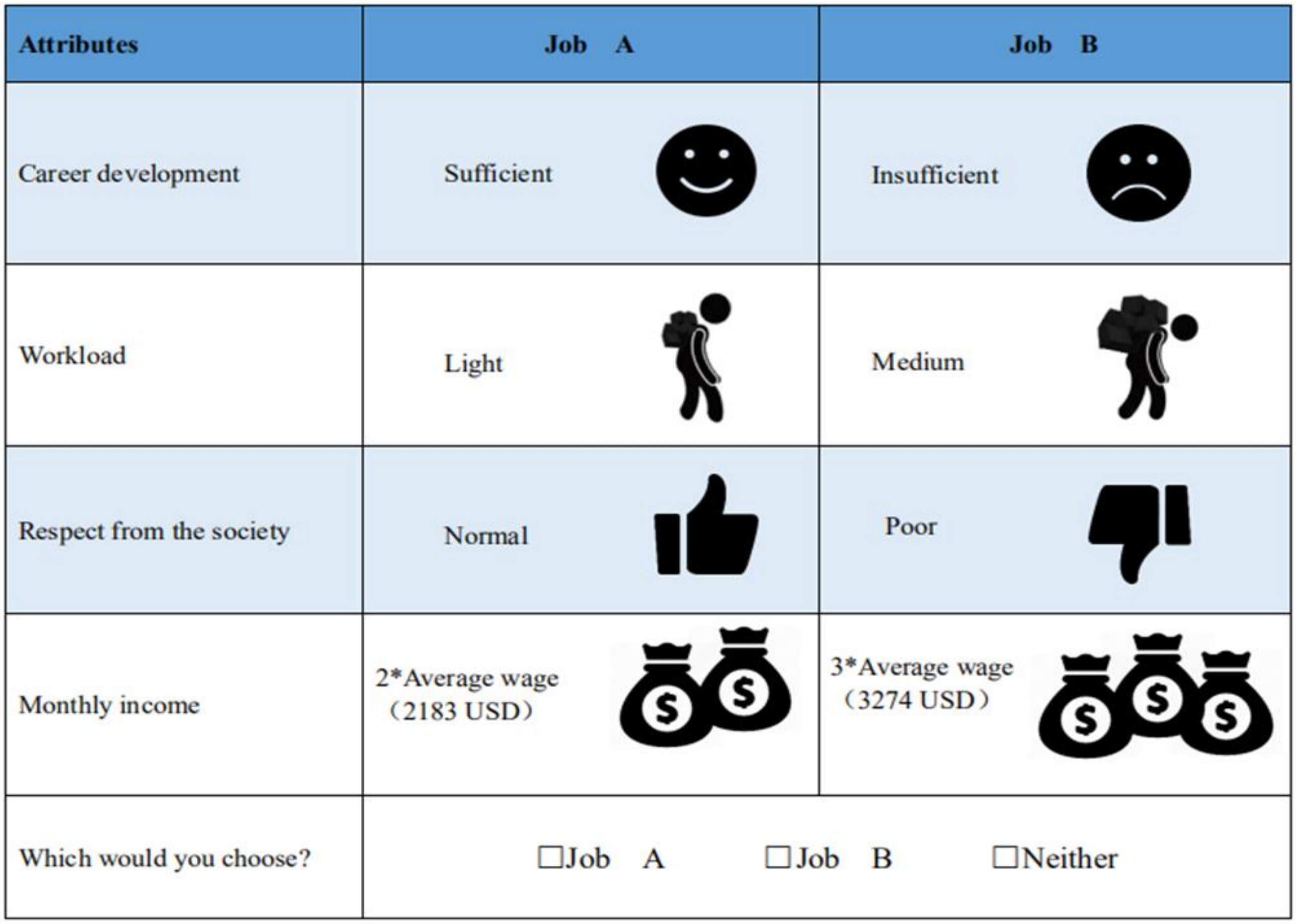

Attributes	Job A	Job B
Career development	Sufficient	Insufficient
Workload	Light	Medium
Respect from the society	Normal	Poor
Monthly income	2*Average wage (2183 USD)	3*Average wage (3274 USD)
Which would you choose?	☐Job A ☐Job B ☐Neither	

Supplementary Figure 1. An example of one choice set used in the DCE
